# Genetic Variations in *ADIPOQ* Gene Are Associated with Chronic Obstructive Pulmonary Disease

**DOI:** 10.1371/journal.pone.0050848

**Published:** 2012-11-28

**Authors:** Yiming Yuan, Haiou Jiang, Jiangying Kuang, Xiaoming Hou, Yulin Feng, Zhiguang Su

**Affiliations:** 1 Department of Geriatrics, West China Hospital, Sichuan University, Chengdu, China; 2 Molecular Medicine Research Center, West China Hospital, and State Key Laboratory of Biotherapy, Sichuan University, Chengdu, China; 3 Department of Respiratory Disease, West China Hospital, Sichuan University, Chengdu, China; University of Pittsburgh, United States of America

## Abstract

**Background:**

Adiponectin is reported to be related to the development of chronic obstructive pulmonary disease (COPD). Genetic variants in the gene encoding adiponectin (*ADIPOQ*) have been reported to be associated with adiponectin level in several genome–wide linkage and association studies. However, relatively little is known about the effects of *ADIPOQ* gene variants on COPD susceptibility. We determined the frequencies of single-nucleotide polymorphisms (SNPs) in *ADIPOQ* in a Chinese Han population and their possible association with COPD susceptibility.

**Methods:**

We conducted a case–control study of 279 COPD patients and 367 age- and gender-distribution-matched control subjects. Seven tagging SNPs in *ADIPOQ*, including rs710445, rs16861205, rs822396, rs7627128, rs1501299, rs3821799 and rs1063537 were genotyped by SNaPshot. Association analysis of genotypes/alleles and haplotypes constructed from these loci with COPD was conducted under different genetic models.

**Results:**

The alleles or genotypes of rs1501299 distributed significantly differently in COPD patients and controls (allele: *P* = 0.002, OR = 1.43 and 95%CI = 1.14–1.79; genotype: *P* = 0.008). The allele A at rs1501299 was potentially associated with an increased risk of COPD in all dominant model analysis (*P* = 0.009; OR: 1.54; 95%CI: 1.11–2.13), recessive model analyses (*P* = 0.015; OR: 1.75; 95% CI: 1.11–2.75) and additive model analyses (*P* = 0.003; OR: 2.11; 95% CI: 1.29–3.47). In haplotype analysis, we observed haplotypes AAAAACT and GGACCTC had protective effects, while haplotypes AGAACTC, AGGCCTC, GGAACTC, GGACACT and GGGCCTC were significantly associated with the increased risk of COPD.

**Conclusions:**

We conducted the first investigation of the association between the SNPs in *ADIPOQ* and COPD risk. Our current findings suggest that *ADIPOQ* may be a potential risk gene for COPD. Further studies in larger groups are warranted to confirm our results.

## Introduction

Chronic obstructive pulmonary disease (COPD) is a major global disease that has been predicted to be the third leading cause of mortality worldwide by the year 2020 [1], and it is estimated to affect nearly 8.2% of the Chinese adult population [2]. Cigarette smoking is the major environmental risk factor for COPD; however, only approximately 15% of smokers develop clinically relevant airflow obstruction [3]_._ The variation in the susceptibility to cigarette smoke, in combination with the familial inheritance pattern of COPD, suggests that there may be a genetic component to the development of COPD [4]. The associations between COPD and polymorphisms in genes with potential importance in COPD pathogenesis have been investigated [5]; however, only α1-antitrypsin has been unequivocally identified as relevant to the development of COPD. Recently, polymorphisms in the CHRNA3-CHRNA5-IREB2, HHIP, and FAM13A loci have been found to be associated with COPD by genome-wide association studies (GWAS) [6]–[8].

There is increasing evidence of systemic inflammation in patients with COPD. Several disease biomarkers have been found to be helpful in assessing systemic and local inflammation, including interleukin (IL) 6, IL-8 and C-reactive protein (CRP) [9], [10]. Adiponectin is a secretary 30 kD protein synthesized by adipocytes in healthy subjects. Its role in inflammation is controversial, as its plasma concentration decreases in diseases such as metabolic syndrome and type II diabetes [11], but increases in some inflammatory diseases such as rheumatoid arthritis and systemic lupus erythematosus [12], [13]. Animal studies, for instance, suggest that reduced expression of adiponectin is associated with development of emphysema and is associated with the pathogenesis of cachexia and osteoporosis [14]. However, human studies indicate that circulating adiponectin levels are raised in COPD and associated with poor health outcomes including increased risk of exacerbations [15]–[17]. Thus, the relationship of adiponectin to COPD outcomes remains uncertain. Genetic factor accounts for about 40–70% of the variation in adiponectin levels [18]. Genetic variants in the gene encoding adiponectin (*ADIPOQ*) have been reported to be associated with adiponectin levels in several genome-wide linkage and association studies [18]–[20]. However, inconsistent findings on the association of genetic variants of *ADIPOQ* with adiponectin levels have been reported [18]–[22], which could be due to a difference in ethnic populations, single nucleotide polymorphism (SNP) selection, and study power.

With these considerations in mind, we hypothesized that polymorphisms in the *ADIPOQ* gene might modulate susceptibility to COPD. To test this hypothesis, we investigated the association of common genetic variants in the *ADIPOQ* gene with the risk of COPD in a Chinese Han population.

## Materials and Methods

### Ethics Statement

The use of human tissue and the protocol in this study were strictly conformed to the principles expressed in the Declaration of Helsinki and were approved by the Ethical Committee of the West China Hospital, Sichuan University, and written informed consent was obtained from all subjects before their participation in the study. The investigator explained the nature, purpose and risks of the study and provided the subject with a copy of the information sheet.

### Subjects

As described previously [23], 279 patients with COPD and 367 age-matched non-COPD control subjects were recruited for this study. The subjects in both groups were unrelated ethnic Han Chinese individuals recruited from Chengdu city or surrounding regions in the Sichuan province of western China. All subjects underwent physical examinations including chest x-ray, anthropometric measurements including body mass index (BMI), assessment of lung function, and blood sampling. The recruitment and the clinical analyses were conducted at the department of respiratory medicine in West China Hospital of Sichuan University; clinical analyses were performed according to the Global Initiative for Chronic Obstructive Lung Disease (GOLD) criteria [24]. COPD patients were enrolled when they suffered from cough, sputum production and dyspnea at least upon exertion and showed chronic irreversible airflow limitation defined by an FEV_1_ (forced expiratory volume in 1s) to FVC (forced vital capacity) ratio <70%, and FEV1 predicted <80% after the inhalation of a *β2*-agonist. Patients were excluded from this study if they had other significant respiratory diseases, such as bronchial asthma, bronchiectasis, lung cancer, or pulmonary tuberculosis based on their chest x-ray test.

The age-matched non-COPD control subjects were volunteers who came to the West China Hospital of Sichuan University for physical examination only. The inclusion criteria for controls were as follows: (1) FEV1/FVC ratio >70%, FEV1% and FVC% predicted >80% and (2) without pulmonary disease. Individuals were excluded if they had a history of chronic lung disease, atopy, an acute pulmonary infection in the 4 weeks before assessment for this study, or a family history of COPD.

### Biochemical Measurements

Blood samples were collected at baseline from patients and controls after an overnight fast. Plasma separated from cells by centrifugation at 500 g for 10 min at room temperature was used for lipid, glucose and adiponectin analyses. The plasma levels of total cholesterol, triglycerides and glucose were determined with an enzymatic kit (Boehringer Mannheim) and calibrated with a plasma calibrator. Circulating total adiponectin level was measured by the enzyme-linked immunosorbent assay method (Quantikine, R&D Systems, Minneapolis, MN, USA).

### SNP Selection and Genotyping

Genotype data of the Chinese population for the *ADIPOQ* region were obtained from the HapMap website (http://www.hapmap.org/), and tag SNPs were selected using the Tagger software implemented in the Haploview software [25], with an r^2^ threshold of 0.8 and minor allele frequencies (MAF) of 0.1. There were seven tagging SNPs (rs710445, rs16861205, rs822396, rs7627128, rs1501299, rs3821799 and rs1063537), which captured all the fifteen SNPs from 3-kb region upstream to 1-kb downstream of the gene (position 188,040,157–188,059,946 bp, GenBank accession number NM_004797.3, NCBI build 36).

Genomic DNA was extracted from peripheral blood leukocytes using a commercial extraction kit (Bioteke Corporation, Beijing, China) according to the manufacturer's instructions. SNPs were genotyped using the ABI SNaPshot method (Applied Biosystems, CA, USA). The genomic regions of interest were amplified by primers shown in Table 1. The PCR products were then purified by incubating with shrimp alkaline phosphatase (SAP) and exo-nuclease I (Exo I). The purified PCR products were used as the templates for SNaPshot reaction using the specific SNaPshot primers (Table 1). 3 µl of pooled PCR products, 1 µl of pooled SNaPshot primers and 1 µl of deionized water were incubated in a GeneAmp 9600 thermal cycler by 25 cycles at 96°C for 10 s, 50°C for 5 s, and 60°C for 30 s, and finally 60°C for 30 s. Then, 1 U of SAP was added to SNaPshot product and incubated at 37°C for an hour to deactivate the enzyme. The SNaPshot reaction products were mixed with Hi-Di formamide and GeneScan-120 LIZ internal size standard (Applied Biosystems), and analyzed on an ABI 3130 Genetic Analyzer (Applied Biosystems, CA, USA). The data were analyzed by the software of GeneMapper 4.0. Genotype analysis was performed in a blinded manner so that the staff was unaware of the cases or control status. For quality control, a 10% masked random sample of cases and controls was tested repetitively by different investigators and all the results were completely concordant.

**Table 1:** Seven SNPs in the *ADIPOQ* gene in the study.

SNPs	Position (build 37.3)	Alleles	Primers (5′-3′)	
			PCR	SNaPshot
rs710445	186,561,518	G/A	ctgaggcaggagaattgtctg	taatgagataaaatgagaaaagcctggcat
			acaaccaggatgcctcagaa	
rs16861205	186,561,634	G/A	cctggcatatagtggcaact	cctgctcgccccagtgagtgctgtttct
			ggatccatgtcctctaacac	
rs822396	186,566,877	A/G	tcatggaacccaggctgatc	ttcccttagggtaggagaaagagatctttattttt
			ggaaacaggaggagagaatc	
rs7627128	186,568,799	C/A	cagagacattcttggagttg	agatgagttggatgtgccacgtgaagagggtagtatagaa
			gacaaacctactcctctgtg	
rs1501299	186,571,123	A/C	cagcaaagccaaagtcttgg	gctttctccctgtgtctaggccttagttaataatgaatg
			tggtgagaagggtgagaaag	
rs3821799	186,571,486	T/C	cca gtggcattcaaccacat	tgtgcaaggctctgttggtggttac
			ccttgaagccttcattcttc	
rs1063537	86,574,075	C/T	gtctccttgagtaccaacag	ttctctcaggagacaataactccagtgatgtt
			atcgcttgaacctgggagg	

### Statistical Analyses

The demographic and clinical data between the COPD patients and the control subjects were compared using the χ2 test and Student’s t-test. A two-sided significance level of α <0.05 was used for all significant tests. Statistical analyses were performed in SPSS version 17.0 and Microsoft Excel. A multiple logistic regression analysis using BMI and glucose as covariates was done to correct the significant *p*-value of adiponectin.

The Hardy-Weinberg equilibrium (HWE) test using two-sided χ2 analysis was done for each SNP among cases and controls. Differences in the distribution of genotypes or alleles under different genetic models (including dominant, recessive and additive models) between the COPD patients and the controls were estimated by using the χ2 test. Odds ratios (OR) and 95% CIs were calculated by unconditional logistic regression analyses [26], [27]. Correction for multiple testing was performed by the SNP spectral decomposition method (SNPSpD) [28]. Under this method, the effective number of independent marker loci (MeffLi) was 7, and the experimental-wide significance threshold to keep type 1 error rate at 5% was 0.0098.

Pairwise linkage disequilibrium (LD) estimation and haplotype reconstruction were performed using SHEsis (http://analysis.bio-x.cn) [29]. For haplotype analysis, only haplotypes with a frequency >3% in at least one group were tested. We also used Haploview 4.2 [25] to estimate LD.

## Results

### General Characteristics of the Subjects

The baseline characteristics, biochemical features and the results of the pulmonary function tests for the 279 patients with COPD and 367 control subjects were presented in Table 2. All patients had FEV_1_ values <80% of predicted and thus were diagnosed with moderate-to-severe COPD according to the Global Initiative for Chronic Obstructive Lung Disease [24] (classification of severity: mild = FEV_1_≥80% of predicted; moderate = FEV_1_≥50% to <80% of predicted; severe = FEV_1_≥30% to <50% of predicted; and very severe = FEV_1_<30% of predicted). The COPD cases and control subjects did not significantly differ in sex, age or smoking history. The FEV1, FEV1/predicted and FEV1/FVC were significantly lower in the COPD patients compared with the controls (*P*<0.01). Compared to control subjects, the COPD patients showed statistically higher glucose concentrations (*p*<0.01), however, they were still within the normal range. In addition, the COPD patients showed a significantly lower BMI (22.03±2.27 kg/m^2^ vs. 23.91±2.48 kg/m^2^, *p*<0.01) and a 1.4-fold higher adiponectin levels (8.54±0.66 µg/ml vs 6.12±0.57 µg/ml; *p*<0.01). The adiponectin levels remained significantly higher in COPD patients after further adjusting for BMI and glucose levels (*p*<0.01).

**Table 2:** Characteristics of COPD patients and control subjects.

Variable	Controls (*n* = 367)	Cases (*n* = 279)	*P*
Age, years	65±8	63±9	NS
Sex (Men/Women)	323/44	239/40	NS
BMI(kg/m^2^)	23.91±2.48	22.03±2.27	<0.01
Total cholesterol(mmol/l)	4.87±0.22	4.96±0.47	NS
Triglycerides(mmol/l)	1.24±0.57	1.19±0.48	NS
Glucose(mmol/l)	5.13±0.12	5.86±0.16	<0.01
Adiponectin(µg/ml)	6.12±0.57	8.54±0.66	<0.01
Smoking history			
0–20 pack years	88	75	NS
≥20 pack years	279	204	NS
FEV1	1.87±0.60	0.97±0.32	<0.01
FEV_1_ of predicted, %	93.7±3.4	46.0±0.4	<0.01
FEV1/FVC, %	78.0±4.6	49.2±8.3	<0.01

Data are presented as means ± SD.

NS, no significant difference (*P*>0.05).

BMI, body mass index.

FEV1, forced expiratory volume in 1 second; FVC, forced vital capacity.

Pack years = (number of cigarettes smoked per day × number of years smoked)/20.

### Distribution of the SNPs in *ADIPOQ* between COPD Patients and Controls

Seven SNPs in *ADIPOQ*, including rs710445, rs16861205, rs822396, rs7627128, rs1501299, rs3821799 and rs1063537, were screened in all 279 patients with COPD and 367 controls using the SNaPshot method. The genotype and allele frequencies of each SNP in both COPD patients and controls were presented in Table 3. All of the tested SNPs didn’t significantly deviate from that expected for a Hardy-Weinberg equilibrium (HWE) in the COPD patients and controls (Table 3, all *P* values were higher than 0.05), illustrating that our subjects presented the source population well.

**Table 3:** Distributions of the *ADIPOQ* SNPs in COPD patients and controls.

SNP	Group	Genotype (freq.%)			*P*	HWE^a^ *P*	Allele (freq.%)		*P*	OR [95%CI]^b^
		GG	AG	AA			G	A		
rs710445	COPD	75(26.9)	147(52.7)	57(20.4)	0.953	0.331	297(53.2)	261(46.8)	0.910	1.01
	Control	102(27.8)	189(51.5)	76(20.7)		0.501	393(53.5)	341(46.5)		[0.81–1.26]
rs16861205		GG	AG	AA			G	A		
	COPD	155(55.6)	112(40.1)	12(4.3)	0.165	0.137	422(75.6)	136(24.8)	0.132	1.22
	Control	230(62.6)	121(33.0)	16(4.4)		0.968	581(79.2)	153(20.8)		[0.94–1.59]
rs822396		AA	AG	GG			A	G		
	COPD	169(60.6)	101(36.2)	9 (3.2)	0.103	0.188	439(78.7)	119(21.3)	0.133	1.24
	Control	249(67.8)	104(28.3)	14 (3.8)		0.451	602(82.0)	132(18.0)		[0.94–1.63]
rs7627128		CC	AC	AA			C	A		
	COPD	119(42.7)	133(47.7)	27 (9.7)	0.998	0.244	371(66.5)	187(33.5)	0.960	1.01
	Control	157(42.8)	175(47.7)	35 (9.5)		0.167	489(66.6)	245(33.4)		[0.80–1.27]
rs1501299		CC	AC	AA			C	A		
	COPD	92(33.0)	139(49.8)	48(17.2)	**0.008**	0.715	323(57.9)	235(42.1)	**0.002**	1.43
	Control	158(43.1)	170(46.3)	39(10.6)		0.499	486(66.2)	248(33.8)		[1.14–1.79]
rs3821799		TT	TC	CC			T	C		
	COPD	110(39.4)	128(45.9)	41(14.7)	0.993	0.705	348(62.4)	210(37.6)	0.970	0.99
	Control	145(39.5)	167(45.5)	55(15.0)		0.544	457(62.3)	277(37.7)		[0.79–1.25]
rs1063537		CC	CT	TT			C	T		
	COPD	131(47.0)	126(45.2)	22 (7.8)	0.823	0.271	388(69.5)	170(30.5)	0.648	1.06
	Control	181(49.3)	157(42.8)	29 (7.9)		0.531	519(70.7)	215(29.3)		[0.83–1.35]

a. HWE: Hardy-Weinberg equilibrium. b. OR: odds ratio; CI: confidence interval.

We compared the differences in frequency distributions of genotypes or alleles of every SNP between COPD patients and controls by χ^2^ test. As shown in Table 3, significant differences in allele or genotype frequencies were observed between COPD patients and controls at rs1501299 (allele: *P* = 0.002, OR = 1.43 and 95%CI = 1.14–1.79; genotype: *P* = 0.008).

### Association of Genotypes with COPD under Different Genetic Models

For each SNP, if one allele frequency is relatively lower compared to another one, it is recognized as the minor allele (Table 3). We assumed that the minor allele of each SNP was a risk allele compared to the wild type allele. We compared the genotype frequencies of every polymorphism between groups under the dominant, recessive and additive genetic models, respectively. As shown in Table 4, the rs1501299 was observed to be associated with COPD risk by all dominant model analysis (*P* = 0.009; OR: 1.54; 95%CI: 1.11–2.13), recessive model analyses (*P* = 0.015; OR: 1.75; 95% CI: 1.11–2.75) and additive model analyses (*P* = 0.003; OR: 2.11; 95% CI: 1.29–3.47).

**Table 4:** Association between *ADIPOQ* SNPs and the risk of COPD under different genetic models.

SNP	Genetic model		*P*	OR [95%CI]
rs710445	Dominant	(AG+AA) vs GG	0.797	1.05 [0.74–1.49]
	Recessive	AA vs.(GG+AG)	0.931	0.98 [0.67–1.45]
	Additive	AG vs. GG	0.765	1.06 [0.73–1.53]
		AA vs. GG	0.932	1.02 [0.65–1.61]
rs16861205	Dominant	(AG+AA) vs GG	0.068	1.34 [0.98–1.84]
	Recessive	AA vs.(GG+AG)	0.971	0.99 [0.46–2.12]
	Additive	AG vs. GG	0.058	1.37 [0.99–1.91]
		AA vs. GG	0.787	1.11 [0.51–2.42]
rs822396	Dominant	(AG+GG) vs. AA	0.055	1.37 [0.99–1.90]
	Recessive	GG vs. (AG+AA)	0.689	0.84 [0.36–1.97]
	Additive	AG vs. AA	0.036	1.43 [1.02–2.00]
		GG vs. AA	0.902	0.95 [0.40–2.24]
rs7627128	Dominant	(AC+AA) vs. CC	0.974	1.01 [0.73–1.38]
	Recessive	AA vs. (CC+AC)	0.952	1.02 [0.60–1.72]
	Additive	AC vs. CC	0.987	1.00 [0.72–1.39]
		AA vs. CC	0.95	1.02 [0.58–1.77]
rs1501299	Dominant	(AC+AA) vs. CC	**0.009**	1.54 [1.11–2.13]
	Recessive	AA vs. (CC+AC)	**0.015**	1.75 [1.11–2.75]
	Additive	AC vs. CC	0.051	1.40 [0.99–1.98]
		AA vs. CC	**0.003**	2.11 [1.29–3.47]
rs3821799	Dominant	(TC+CC) vs TT	0.983	1.00 [0.73–1.38]
	Recessive	CC vs. (TT+TC)	0.918	0.98 [0.63–1.52]
	Additive	TC vs. TT	0.952	1.01 [0.72–1.42]
		CC vs. TT	0.942	0.98 [0.61–1.58]
rs1063537	Dominant	(CT+TT) vs. CC	0.551	1.01 [0.81–1.50]
	Recessive	TT vs. (CC+CT)	0.994	1.00 [0.56–1.78]
	Additive	CT vs. CC	0.533	1.11 [0.80–1.53]
		TT vs. CC	0.877	1.05 [0.58–1.91]

OR: odds ratio; CI: confidence interval.

### rs1501299 is Associated with Plasma Adiponectin Levels

**Figure 1:**
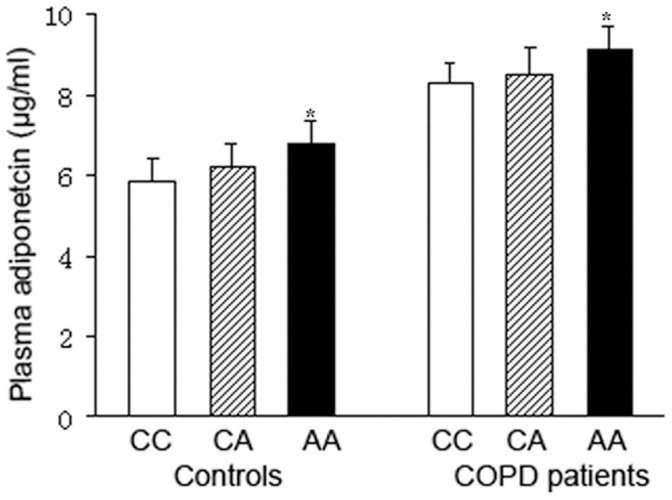
The allele effect of rs1501299 on plasma adiponectin levels. Values were expressed as means ± SD. *P* **<**0.05 vs. subjects with CC genotype.

We investigated the relationship between rs1501299 and plasma adiponectin levels in both COPD patients and control subjects (Figure 1). Compared to subjects with homozygote CC at rs1501299, the subjects with homozygote AA had significantly higher adiponectin levels after adjusting for BMI and glucose levels (6.82±0.52 µg/ml vs. 5.85±0.57 µg/ml in controls, 9.13±0.49 µg/ml vs. 8.27±0.53 µg/ml in COPD patients, *P* **<**0.05 in both groups).

### Linkage Disequilibrium (LD) between SNPs and Haplotype Analysis

**Figure 2:**
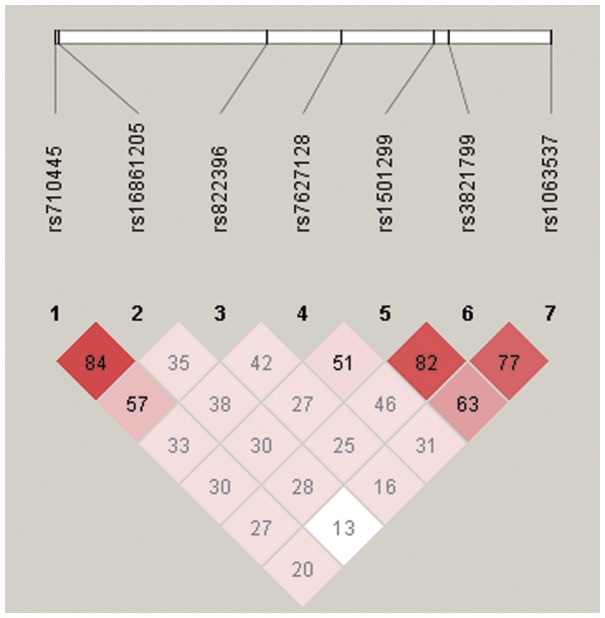
Linkage disequilibrium (LD) plots for *ADIPOQ*. The LD plots were generated by Haploview 4.2. Polymorphisms are identified by their dbSNP rs numbers, and their relative positions are marked by vertical lines within the white horizontal bar. The numbers within squares indicate the D’ value, expressed as a percentile.

The extent of linkage disequilibrium in pairwise combinations of alleles in different SNP was estimated by means of maximum likelihood from the genotype frequency in the COPD and control groups. Pairwise LD between the seven SNPs was shown in Table 5 and Figure 2. Based on LD determinations, two blocks with moderate LD were detected: block 1 is composed of rs710445 and rs16861205, block 2 is composed of rs1501299, rs3821799 and rs1065537.

**Table 5:** Pairwise linkage disequilibrium analysis of SNPs of the *ADIPOQ* gene.

D'	rs16861205	rs822396	rs7627128	rs1501299	rs3821799	rs1063537
rs710445	**0.846**	0.573	0.337	0.308	0.277	0.203
rs16861205	–	0.358	0.380	0.302	0.284	0.130
rs822396	–	–	0.424	0.273	0.250	0.160
rs7627128	–	–	–	0.511	0.467	0.318
rs1501299	–	–	–	–	**0.820**	0.639
rs3821799	–	–	–	–	–	**0.775**

D': linkage disequilibrium coefficient.

We estimated the frequencies of haplotypes constructed from phased multi-locus genotypes in *ADIPOQ*. The haplotypes with a frequency higher than 3% in at least one group were involved in the haplotype analysis (Table 6). The global result for block 1 (rs710445 and rs16861205) was: χ^2^ = 22.99 while df = 3, *P* = 4.16×10^−5^. The global result for block 2 (rs1501299, rs3821799 and rs1065537) was: χ^2^ = 79.33 while df = 5, *P* = 7.66×10^−15^. The overall frequency distribution of haplotype composed of all seven SNPs was significantly different between cases and controls (total global χ^2^ = 159.35 while df = 10, *P* = 4.40×10^−29^).

**Table 6:** Frequencies of pairwise haplotype constructed by SNPs in *ADIPOQ*.

Block	Haplotype^a,b^	freq (case)	freq(control)	χ^2^	Fisher's *P*	OR [95%CI]^c,d^
1	AA	0.204	0.213	0.16	0.686	0.95 [0.72–1.24]
	AG	0.264	0.252	0.26	0.613	1.07 [0.83–1.37]
	GA	0.040	0.004	22.14	**2.61×10^−6^**	11.54 [3.20–41.57]
	GG	0.492	0.532	1.98	0.160	0.85 [0.69–1.06]
	Global			22.99	**4.16×10^−5^**	
2	ACC	0.068	0.128	12.40	**4.33×10^−4^**	0.50 [0.34–0.74]
	ACT	0.279	0.193	13.19	**2.85×10^−4^**	1.62 [1.25–2.10]
	ATC	0.064	0.017	18.87	**1.43×10^−5^**	3.85 [2.01–7.37]
	CCT	0.014	0.032	4.48	0.054	0.42 [0.19–1.06]
	CTC	0.552	0.537	0.21	0.645	1.05 [0.84–1.32]
	CTT	0.002	0.068	36.16	**1.97×10^−9^**	0.02 [0.01–0.12]
	Global			79.33	**7.66×10^−15^**	
Total	AAAAACT	0.014	0.032	6.47	**0.011**	0.36 [0.16–0.82]
	AAGAACT	0.040	0.038	0.12	0.725	0.90 [0.51–1.60]
	AGAAACT	0.025	0.037	2.91	0.088	0.56 [0.29–1.10]
	AGAACTC	0.037	0.012	6.76	**0.009**	2.80 [1.25–6.27]
	AGACCTC	0.078	0.064	0.06	0.802	1.06 [0.68–1.64]
	AGGCCTC	0.051	0.005	22.74	**1.91×10^−6^**	8.73 [3.02–25.20]
	GGAAACT	0.048	0.023	3.76	0.053	1.84 [0.99–3.43]
	GGAACTC	0.050	0.010	15.98	**6.48×10^−5^**	4.76 [2.06–11.00]
	GGACACT	0.092	0.008	46.57	**9.89×10^−12^**	12.03 [4.96–29.21]
	GGACCTC	0.212	0.359	72.32	**4.00×10^−15^**	0.29 [0.22–0.39]
	GGGCCTC	0.034	0.000	21.95	**2.87×10^−6^**	–
	Global			159.35	**4.40×10^−29^**	

a. The order of SNPs from left to right is: rs710445 and rs16861205 for block 1; rs1501299, rs3821799 and rs1063537 for block 2; rs710445, rs16861205, rs822396, rs7627128, rs1501299, rs3821799 and rs1063537 for total.

b. Only haplotypes with a frequency >3% in at least one group were listed.

c. OR: odds ratio; CI: confidence interval.

d. The OR could not be calculated for the haplotype GGGCCTC, because of the zero value in the population.

The results of the association between the *ADIPOQ* haplotype and the risk of COPD were listed in Table 6. Haplotype GA in block 1 was found to be associated with an increased risk of COPD (OR = 11.54, 95%CI = 3.20–41.57, *P* = 2.61×10^−6^). In block 2, two haplotypes were observed to be associated with the risk of COPD (ACT: OR = 1.62, 95%CI = 1.20–2.10, *P* = 2.85×10^−4^; ATC: OR = 3.85, 95%CI = 2.01–7.37, *P* = 1.43×10^−5^), while haplotypes ACC and CTT were protective from COPD (ACC: OR = 0.50, 95%CI = 0.34–0.74, *P* = 4.33×10^−4^; CTT: OR = 0.02, 95%CI = 0.01–0.12, *P* = 1.97×10^−9^). Global haplotype association analyses showed that five haplotypes, including AGAACTC, AGGCCTC, GGAACTC, GGACACT and GGGCCTC, were significantly associated with the risk of COPD (all OR>1.00, and *P*<0.01). In addition, two protective haplotypes AAAAACT (OR = 0.36, 95%CI = 0.16–0.82, *P* = 0.011) and GGACCTC (OR = 0.29, 95%CI = 0.22–0.39, *P* = 4.00×10^−15^), which were associated with a decreased risk of COPD.

## Discussion

Adiponectin, an adipose tissue-derived cytokine, has important roles in insulin sensitization, cardioprotection, and anti-inammatory processes. Plasma adiponectin level is negatively correlated with body mass index (BMI), glucose, insulin and triglyceride levels and is positively associated with high-density lipoprotein cholesterol (HDL-C) concentration and insulin-stimulated glucose disposal [30]. Although its action on the respiratory system is not fully known, expression of adiponectin and its functional receptors on airway epithelium have been reported [31]. There have been several clinical studies reporting on the relationship between circulating adiponectin and COPD, and elevation of plasma adiponectin level was found in patients with stable and acute exacerbation of COPD [15]–[17], [32]. The most bioactive form of adiponectin is a 400-kDa high molecular weight (HMW) complex [33], which was also found to be increased dramatically in COPD patients in a recent report [34].

In this case-control study in a Han Chinese population, we evaluated the possible association of *ADIPOQ* polymorphisms with susceptibility to COPD. To the best of our knowledge, this study was the first investigation of the association between the SNPs in *ADIPOQ* and COPD risk. Our current findings suggested that rs1501299 associated with the risk of COPD. In comparison with allele G at rs1501299, the allele A could increase the risk of COPD under all dominant, recessive and additive genetic models. The polymorphism rs1501299, also known as C276A, in *ADIPOQ* has been found to be in association with adiponectin levels in diverse population [35]–[37], and its A allele is associated with higher adiponectin. One report showed that serum adiponectin levels were higher in COPD patients than in control subjects and were associated with weight loss and systemic inflammation as assessed by circulating tumor necrosis factor-α (TNF-α) levels [15]. A recently study showed that serum adiponectin was associated with all-cause mortality in COPD patients [38]. However, this association is conflicted in animal models. In one study, hypoadiponectinemia in *ADIPOQ* gene-knockout mice was associated with emphysema-like changes in the lungs and extrapulmonary features including systemic inflammation, obesity, and osteoporosis [14], while another study reported the opposite effect in which mice deficient in adiponectin were protected from emphysema when they were exposed to cigarette smoke [39]. In our current study, the association of rs1501299 in *ADIPOQ* and COPD might be modulated by its effects on the adiponection levels. However, rs1501299 is in intron 2 of the *ADIPOQ* gene and does not have a known function. It is probably a marker of some other variant affecting adiponectin expression. We assessed the extent of linkage disequilibrium (LD) between rs1501299 and other SNPs in exons, 3'UTR and promoter regions of the *ADIPOQ* gene in Han Chinese in Beijing (CHB) using HapMap project data (Figure S1), which indicated that rs1501299 was in a LD block encompassing the whole 3'UTR of the *ADIPOQ* gene, but whether and how far such block extends beyond the gene boundaries remain to be determined [40].

In addition to the genotype analysis, our study also adopted a haplotype based approach. Haplotype analysis, in which several SNPs within the same gene are evaluated simultaneously, can provide more information than a single SNP and thus elevates the statistical power of the analysis [41]. Using this approach, we provided strong support that *ADIPOQ* variations contributed to the susceptibility to COPD. LD analysis showed that some SNPs in *ADIPOQ* gene were in moderate LD, and some haplotypes with low frequency were found to affect the risk of COPD dramatically. Haplotypes AAAAACT and GGACCTC were associated with a reductive risk of COPD, while haplotypes AGAACTC, AGGCCTC, GGAACTC, GGACACT and GGGCCTC increased the risk of developing COPD, indicating the complexity of *ADIPOQ* gene in the development of COPD. This might be attributable to the complex genetic determinants of plasma adiponectin levels. In addition to the SNP in *ADIPOQ* gene, other gene loci such as 14q13 have been reported to affect plasma adiponectin value and play a much bigger role [40]. Recently, Genome wide association study (GWAS) for genetic markers in determining plasma adiponectin value in Korean population reported that genetic variants in *CDH13* on chromosome 16, but not genetic variants in the *ADIPOQ* gene, influence adiponectin levels [42]. However, another GWAS using plasma adiponectin as a quantitative trait demonstrated the *ADIPOQ* gene as the only major gene for plasma adiponectin in Caucasian population [20].

We are aware that the significant results in this study could prove to be false positives because of the relatively small sample size. 279 COPD patients and 367 control subjects were not relatively large among COPD association studies published to date, further studies using larger populations are needed. But even with a larger sample, the functional and biological impacts of the described polymorphisms would require further study. Possible gene-gene and gene-environment interactions also pose a challenge for genetic analysis of COPD association studies. We selected SNPs with MAF higher than 10% in the Han Chinese population (CHB) using HapMap project data, this is not suited for situations where genetic architecture is such that multiple rare disease-causing variants contribute significantly to disease risk. Recent studies demonstrate that identification of rare variants may lead to critically important insights about disease etiology through implication of new genes and/or pathways [43], [44]. The rare variants in the *ADIPOQ* gene should be investigated to clarify their susceptibility to the development of COPD.

In conclusion, our comprehensive analysis of SNPs in the *ADIPOQ* gene suggests that *ADIPOQ* genotypes and haplotypes are associated with COPD risk. These findings indicate that the genetic variants of *ADIPOQ* gene play a complex role in the development of COPD, and that interactions of loci in *ADIPOQ* gene may be more important than a single locus. Our findings in this study provided new evidence for the association between SNPs and haplotypes of *ADIPOQ* gene and the risk of COPD.

## Supporting Information

Figure S1 **Relative position of SNPs and LD map for** ***ADIPOQ*** **in the Han Chinese population (CHB) using HapMap project data.** This figure shows that strong LD was observed between rs1501299 and the SNPs in the 3′UTR of *ADIPOQ*.
(TIF)
