# Supplementary figures and images for: Genetic Variations in ADIPOQ Gene Are Associated with Chronic Obstructive Pulmonary Disease
Source: PLoS One. 2012 Nov 28;7(11):e50848. doi: 10.1371/journal.pone.0050848 (PMC3508992; doi:10.1371/journal.pone.0050848)

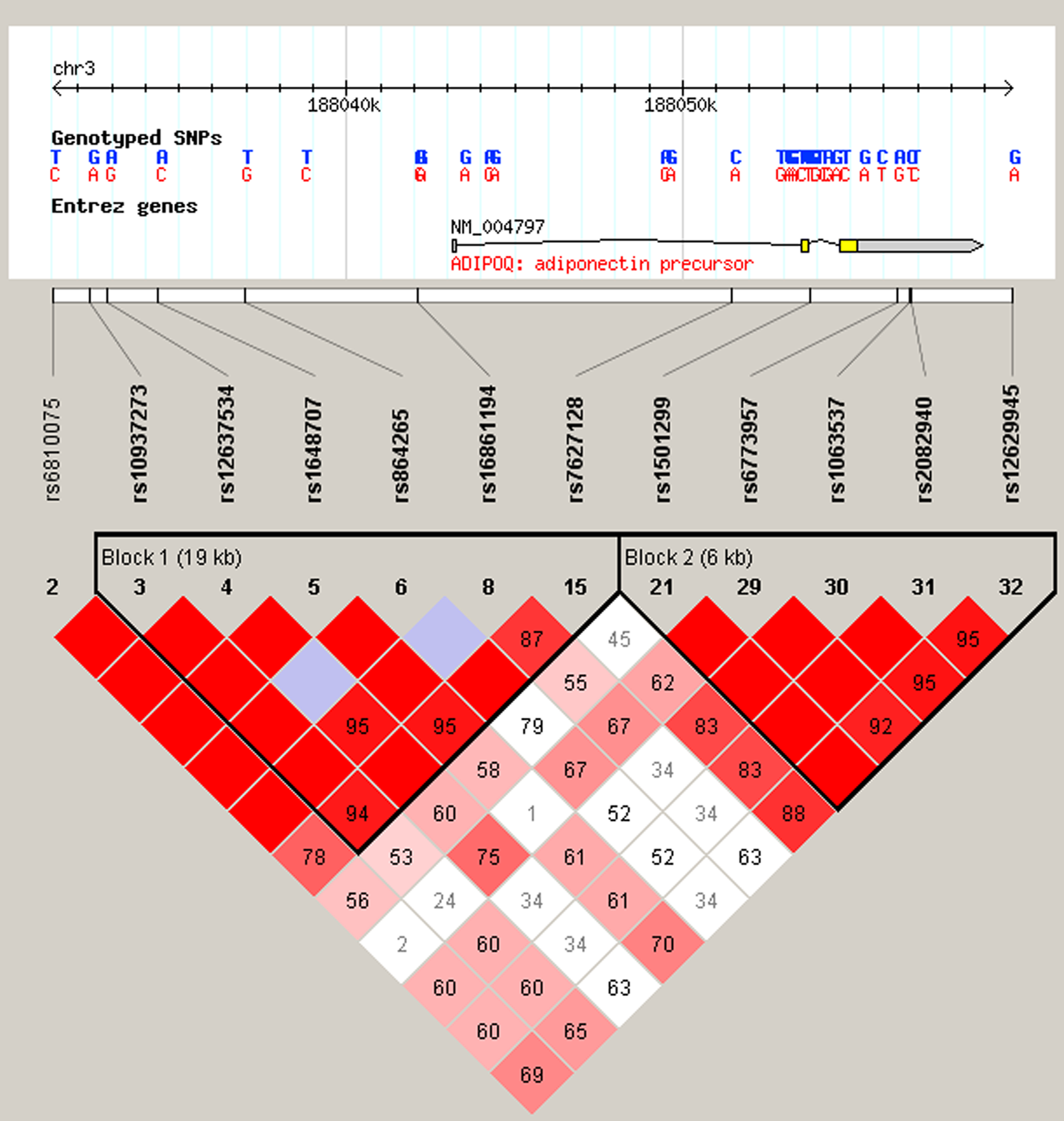

Supplement: Figure S1 — Relative position of SNPs and LD map for ADIPOQ in the Han Chinese population (CHB) using HapMap project data. This figure shows that strong LD was observed between rs1501299 and the SNPs in the 3′UTR of ADIPOQ. (TIF) [file pone.0050848.s001.tif]
